# Octopamine Regulates Antennal Sensory Neurons via Daytime-Dependent Changes in cAMP and IP_3_ Levels in the Hawkmoth *Manduca sexta*

**DOI:** 10.1371/journal.pone.0121230

**Published:** 2015-03-18

**Authors:** Thomas Schendzielorz, Katja Schirmer, Paul Stolte, Monika Stengl

**Affiliations:** University of Kassel, Biology, Animal Physiology, 34132, Kassel, Germany; University of California, Los Angeles, UNITED STATES

## Abstract

The biogenic amine octopamine (OA) mediates reward signals in olfactory learning and memory as well as circadian rhythms of sleep and activity. In the crepuscular hawkmoth *Manduca sexta*, OA changed pheromone detection thresholds daytime-dependently, suggesting that OA confers circadian control of olfactory transduction. Thus, with enzyme-linked immunosorbent assays we searched hawkmoth antennae for daytime-dependent changes in the concentration of OA and its respective second messengers. Antennal stimulation with OA raised cAMP- and IP_3_ levels. Furthermore, antennae expressed daytime-dependent changes in the concentration of OA, with maxima at Zeitgebertime (ZT) 20 when moths were active and also maximal concentrations of cAMP occurred. Maximal IP_3_ levels at ZT 18 and 23 correlated with maximal flight activity of male moths, while minimal IP_3_ levels at dusk correlated with peaks of feeding activity. Half maximal effective concentration (EC_50_) for activation of the OA-receptor decreased during the moth’s activity phase suggesting daytime-dependent changes in OA receptor sensitivity. With an antiserum against tyramine, the precursor of OA, two centrifugal neurons were detected projecting out into the sensory cell layer of the antenna, possibly mediating more rapid stimulus-dependent OA actions. Indeed, in fast kinetic assays OA receptor stimulation increased cAMP concentrations within 50 msec. Thus, we hypothesize that fast, stimulus-dependent centrifugal control of OA-release in the antenna occurs. Additional slow systemic OA actions might be based upon circadian release of OA into the hemolymph mediating circadian rhythms of antennal second messenger levels. The resulting rhythms of odor sensitivity are suggested to underlie circadian rhythms in odor-mediated behavior.

## Introduction

Sex-pheromone release by females as well as pheromone-dependent mating flight of males express synchronized circadian rhythms in various insects like the hawkmoth *Manduca sexta* [1–7]. However, location and mechanisms of the respective circadian pacemakers and the circadian coupling signals which synchronize male and female mating behaviour are mostly unknown. In the hemolymph of *Trichoplysia ni* a circadian rhythm was detected in the concentration of the biogenic amine octopamine (OA), which peaked during the moths’ activity phase [2]. The insect neurotransmitter, neuromodulator and neurohormone OA is a functional homolog of adrenergic transmitters in vertebrates. It promotes wakefulness, controls diverse physiological processes and behavioral responses, and prepares the insect for actions with high energy demand [8–10]. In addition, OA mediates olfactory learning at the level of the antennal lobe and the mushroom bodies and substitutes for the appetitive reward [11–17]. Furthermore, OA not only affects central processing of odor-dependent behaviour it also modulates the sensory periphery by reducing response thresholds and reversing adaptation [18–27].

Also in *M*. *sexta* application of OA sensitized/disadapted pheromone responses of olfactory receptor neurons (ORNs) [24]. Since OA actions differed Zeitgebertime (ZT)-dependently in the hawkmoth, either the amount of OA concentrations already present in the antenna differed, or ORNs expressed circadian rhythms of OA-sensitivity. Thus, we hypothesized that a circadian rhythm in OA concentration in the hemolymph of the hawkmoth mediates circadian changes of pheromone detection thresholds at the sensory periphery. Since pheromone detection in the hawkmoth involves gating of second messenger-dependent ion channels we hypothesized that OA regulates pheromone response thresholds via modulation of second messenger levels in ORNs [28,29]. To test this hypothesis in *M*. *sexta*, enzyme-linked immunosorbent assays (ELISAs) were employed determining daytime-dependent changes in baseline concentrations of OA, cAMP, cGMP, and IP3 in hawkmoth’ antennae as a possible basis for daily rhythms in odor detection thresholds. Since OA activates G-protein coupled receptors [26], with ELISAs OA effects on cAMP- and IP_3_- synthesis were analyzed. Furthermore, it was examined whether the sensitivity of OA receptors varied daytime-dependently by calculating the average effective dose (EC_50_) of OA during rest- (ZT 9) and activity phases (ZT 20). Since next to slow, systemic actions of OA over the course of minutes to hours, OA also confers acute stress responses on the time course of ms, we examined the kinetics of OA receptor signalling with high temporal resolution. In addition, with immunocytochemistry employing an antibody against the OA precursor tyramine we searched for centrifugal aminergic neurons branching directly in the antenna. Indeed, our data suggest that OA plays a dual role in the antenna, as a slow, hemolymph-born circadian coupling signal setting daytime-dependent thresholds of sensory neurons during the activity and wake cycle and as a modulator of olfactory transduction in response to acute stress signals via centrifugal octopaminergic neurons.

## Materials and Methods

### Animals

For all experiments adult male *M*. *sexta* were raised in 17:7 hour long-day photoperiods (approximately 500 lux) including one hour dusk and dawn (approximately 50 lux), at 40 to 55% relative humidity and 26°C temperature in a 19 m^3^ flight room. The hawkmoths were raised from eggs and the larvae were fed with an artificial diet (modified after [30]). A few days before eclosion male pupae were cleaned from pheromone with alcohol and isolated in a separate flight room without females. The adult animals were fed with Colibri-nectar (*Nektar-plus*, www.nectar.com, London, England) which was presented in cups wrapped in artificial flowers measuring 6.5 cm in diameter and scented with bergamot oil [31].

### Quantification of octopamine

For sample collections three male hawkmoths each were taken out of the flight room at ZT 1, 9, 16, 18, 20, and 23. Then, the animals were quickly shock-frozen in liquid nitrogen, their antennae were ground in a mortar and transferred into cups. To disrupt enzymatic reactions nitrogen cooled samples were mixed with 100 μl 7% perchloric acid followed by 250 μl 10 mM ethylenediaminetetraacetic acid (EDTA) solution. Then, the mixture was centrifuged at 900 g for 15 minutes at 4°C. For neutralization 275 μl supernatant was mixed in 400 μl 10 mM EDTA as well as 400 μl chloroform/trioctylamine solution (1:1) and centrifuged at 500 g for 5 minutes at 4°C. OA quantification was performed in triplicate with a commercially available OA kit (MBS726911, Mybiosource, San Diego, USA).

### Behavioral analysis

To compare biochemical and behavioral data, flight and feeding activity of isolated adult male hawkmoths were detected by visual observation in flight rooms. Only male hawkmoths were kept in this room (~19 m^3^) with 17:7 hour long-day photoperiods (approximately 500 lux) including one hour dusk and dawn (approximately 50 lux), at 40 to 55% relative humidity and 26°C temperature. Since never any female was allowed to this room it should not be contaminated with pheromones. Due to variation in number of animals in this room (total observed n = 127), launching and feeding events per day and hour were noticed and normalized by dividing number of events per hour by total number of events per day (100%).

### Preparation and quantification of second messengers

Samples were collected as described previously for OA quantification at ZT 1, 9, 16, 18, 20, and 23. Homogenization, incubation, neutralization, and normalization were performed as described in Schendzielorz *et al*. (2012). All incubation buffers contained 10 nM free calcium. For quantifying cAMP- and cGMP concentrations 1 mM 3-isobutyl-1-methylxanthine (IBMX) was used to reduce cyclic nucleotide degradation. OA, forskolin (FSK), m-3M3FBS, and epinastine (EPI) were used in incubation buffers to analyze effects on second messenger synthesis. Commercially available immunoassay kits for determining cAMP-, cGMP-, and IP_3_ concentrations were employed (581001, 581021, Cayman, Michigan, USA; CSB-E12636h, Cusabio, Wuhan, P.R. China).

### Rapid kinetic-assays

To determine whether OA-signalling takes place in the ms range rather than in the range of seconds to minutes, assays for rapid kinetic measurements were developed. In a self-made setup computer-controlled pressure injections mixed respective incubation buffers with antennal homogenates. The reaction was stopped with injection of 7% perchloric acid solution after defined delays in the ms range. Pressure injection of reagents was controlled via magnetic valves. Neutralization, normalization, and second messenger quantification was performed as described previously [29].

### Immunocytochemistry

Since commercially available OA-antibodies did not work in the hawkmoth, instead we employed immunocytochemistry with antibodies against tyramine, the precursor of OA. Before dissecting the antennae of the head animals were anaesthetized (see [32]). Then, the flagella of the antennae were cut into five annuli each and fixed for two hours at room temperature in 2.5% glutaraldehyde, 1% Triton X-100 (TrX), and sodium metabisulfite (0.1 M) in sodium phosphate buffer (PB, 0.1 M, pH 7.4). To protect the tissue against damage by frost, antennae were incubated in 30% sucrose overnight at 4°C. Then, antennae were embedded in gelatine/albumin (4.8% gelatin, 20% ovalbumin in distilled water) and postfixed overnight at 4°C in 10% formalin in PB. Thereafter, the antennae were shock-frozen in isopentane at -130°C and sectioned (20 μm) longitudinally with a cryostat (CM3050 S, Leica, Wetzlar, Germany). The free-floating sections were incubated for 10 minutes at room temperature in PB containing 0.1 M sodium borohydride and 0.1% TrX. Afterwards, sections were preincubated in a blocking solution containing 10% Roti-block (Carl Roth, Karlsruhe, Germany), 2% normal goat serum (Dianova, Hamburg, Germany), 5% TrX, and 0.5 M sodium chloride in PB overnight at 4°C. All further incubation steps were carried out in this blocking solution. Primary antibody, polyclonal rabbit anti-tyramine (TA; Chemicon-Millipore, Bedford, USA; 1:15,000) was incubated for at least 18 hours at 4°C. Secondary antibody (peroxidase conjugated goat-anti rabbit, Dianova) was used at a dilution of 1:300 and incubated for two hours at room temperature. The immunoperoxidase labelled sections were subsequently treated with a solution of 0.03% 3,3’-diaminobenzidine tetrahydrochloride (Sigma-Aldrich, Munich, Germany), 0.015% H_2_O_2_, and 0.6% Nickel(II)sulfate-hexahydrate in Tris-buffered saline (0.55 M Tris-HCl, pH 7.6) for 5 minutes. Sections were mounted on chromalum/gelatine coated microscope slides.

### Data analysis

Before data were evaluated statistically, the distributions were analysed by Shapiro Wilk tests. Since data did not display normal distributions the nonparametric Kruskal-Wallis- and Dunn’s post hoc tests were employed for data analysis. Arithmetic means and standard errors of data were calculated and are stated in the text and figures.

## Results

In the male hawkmoth *M*. *sexta* ELISAs and rapid kinetic assays with antennal tissue were performed to analyze signalling of OA at the sensory periphery. Since OA sensitized pheromone responses daytime-dependently [24] it was examined whether daytime-dependent oscillations of antennal baseline concentrations of OA occur. Furthermore, it was searched for daytime-dependent oscillation in baseline levels of cAMP, IP_3_, and/or cGMP concentrations as possible direct or indirect effectors of OA-receptor-signalling. Finally, after examination of OA-signalling with ELISAs at different time scales, with immunocytochemistry it was determined whether aminergig centrifugal neurons branch in the antenna as possible mediators of stress stimulus- or reward-dependent control of antennal sensitivity.

### Antennal OA concentrations vary daytime-dependently and are maximal during the moth's activity phase

**Fig 1:**
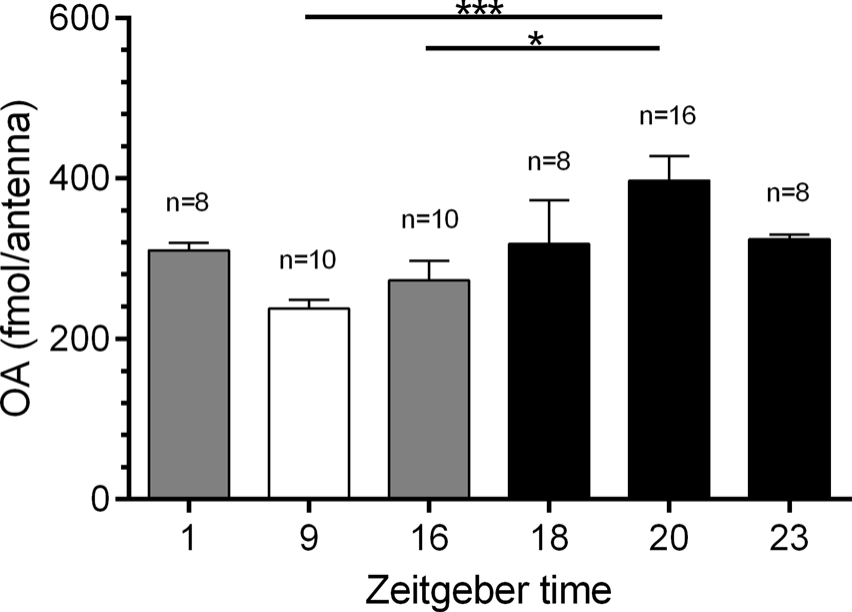
Octopamine (OA) concentrations vary Zeitgebertime (ZT)-dependently in antennae of *Manduca sexta* males. With ELISAs maximum concentrations in OA levels were found at ZT 20 (397.4 fmol/antenna, n = 16), the hawkmoths’ activity phase, while minimal levels occurred at the resting phase (ZT 9, 237.4 fmol/antenna, n = 10, Dunn’s post hoc test, * p < 0.05). n = 1: mean of 3 male antennae.

**Fig 2:**
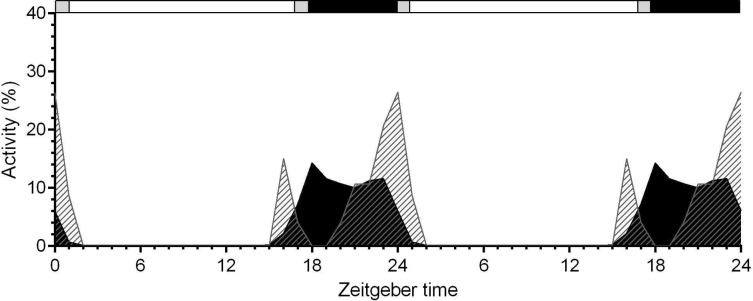
Hawkmoth express maximal feeding (grey stripes) at dusk and dawn and maximal flight activity (black) at night. Data represents mean of seven observations (n = 127 animals) over each 24 hours (double plotted). Upper bar: open bar = light phase; grey bar = low light levels at dusk and dawn (Material and Methods); closed bar = dark phase.

Quantification of OA concentrations of single male antennae at ZT 1, 9, 16, 18, 20, and 23 revealed a significant difference of OA concentrations between all ZTs tested (Kruskal-Wallis test, p < 0.05; Fig. 1, Table 1). The lowest OA concentration was measured at ZT 9 (237.4 fmol/antenna, n = 10) and the highest at ZT 20 (397.4 fmol/antenna, n = 16). OA levels at ZT 9 and ZT 20 as well as at ZT 16 (272.9 fmol/antenna, n = 10) and ZT 20 differed significantly (Dunn’s post hoc test; p < 0.05). To search for correlations between OA concentrations and changes in the hawkmoth's activity feeding and flight behaviour of isolated male hawkmoths (without females) were observed over the course of the day (Fig. 2). Scented artificial flowers with sugar water were offered continuously for feeding, while the males were isolated from the females and, thus, were not exposed to female pheromones. Nevertheless, feeding activity was biphasic with a maximum at dusk and one at dawn. Also, flight activity seemed to be biphasic and daytime-dependent. It was restricted to the night with one maximum at ZT 18 and another, smaller at ZT 23. Thus, maximal OA levels were detected in the middle of the hawkmoths' activity phase (Fig. 2).

**Table 1:** Quantification of octopamine amount per antenna as well as antennal second messenger concentration.

	Octopamine (fmol/antenna)		cAMP (pmol/mg)		IP_3_		cGMP (pmol/mg)	
					(fmol/mg)			
	mean±SE	n	mean±SE	n	mean±SE	n	mean±SE	n
ZT 1	310.4±8.8	8	66.5±11.1	10	389.8±79.3	10	4.6±2.0	5
ZT 9	237.4±10.9	10	71.5±6.5	21	333.9±51.7	32	7.1±1.0	20
ZT 16	272.9±24.6	10	84.9±23.1	10	291.5±39.1	16	6.5±1.2	10
ZT 18	318.2±54.5	8	113.9±33.4	9	565.2±77.9	16	7.3±1.4	14
ZT 20	397.4±30.1	16	151.5±19.5	34	408.3±20.6	33	6.7±0.7	23
ZT 23	324.0±6.0	8	83.0±21.4	11	484.3±75.6	9	6.3±2.2	6

### The concentrations of cAMP and IP_3_ vary daytime-dependently

**Fig 3:**
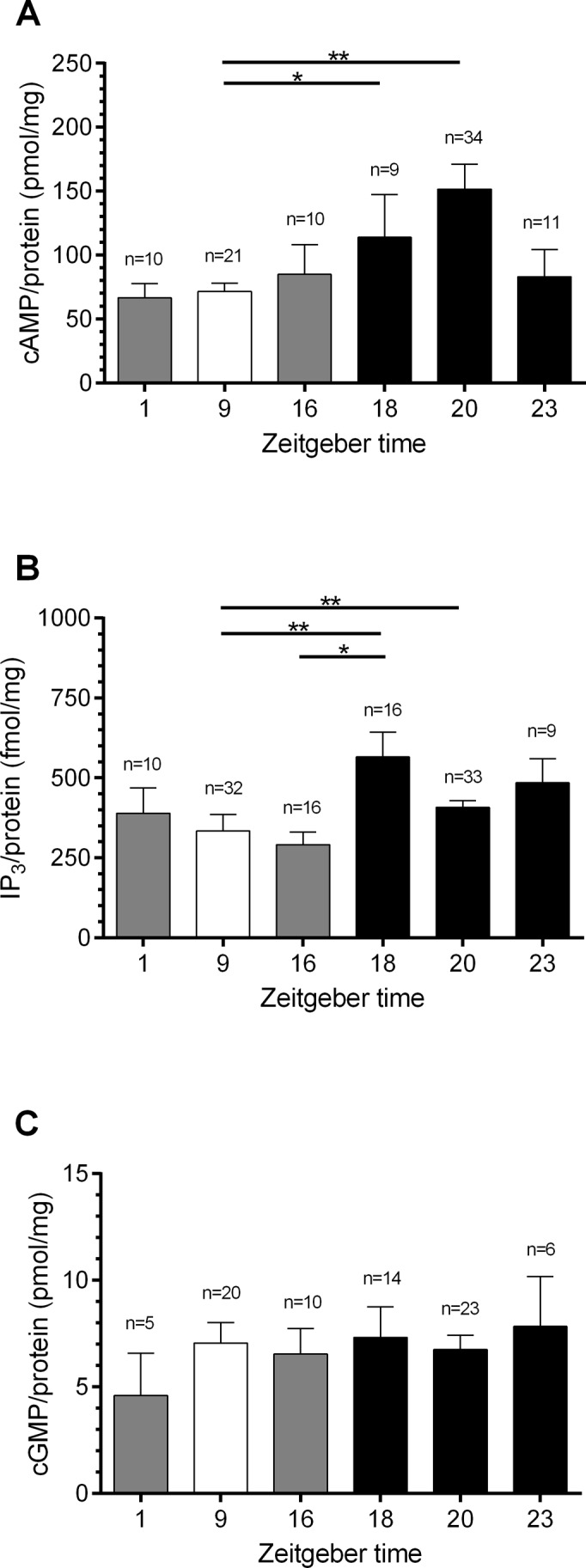
Hawkmoth antennae show Zeitgebertime (ZT)-dependent changes in cAMP- (A) and IP_3_- (C), but not in cGMP (B) baseline levels (n = 1 contains 6 antennae). **A:** The maximum in cAMP concentration measured with ELISAs at ZT 20 (151.5 pmol/mg, n = 34) and the minimum at ZT 9 (71.5 pmol/mg, n = 21) differ highly significant from each other (Dunn’s post hoc test, ** p < 0.01). **B:** The IP_3_ level peaked at ZT 18 (565.2 fmol/mg, n = 16) and was minimal at ZT 16 (291.5 fmol/mg, n = 16, Dunn’s post hoc test, * p < 0.05). **C:** cGMP levels remained constant during the day. Open columns = light phase; grey columns = dusk and dawn; closed columns = dark phase.

To determine whether daytime-dependent changes in OA might result in daytime-dependent changes in second messenger levels male hawkmoth antennae were collected under conditions of isolation without exposure to female pheromones and were processed for ELISAs. Indeed, daytime-dependent changes were observed in cAMP- and IP_3_- (Fig. 3A,B, Table 1), but not in cGMP concentrations (Fig. 3C). A highly significant difference in cAMP- and IP_3_ baseline levels was detected at ZT 1, 9, 16, 18, 20, and 23 (Kruskal-Wallis test, p < 0.001). The lowest cAMP concentrations were found at ZT 1 (66.5 pmol/mg; n = 10) and ZT 9 (71.5 pmol/mg; n = 10), while the maximum was measured at ZT 20 (151.5 pmol/mg; n = 34). At ZT 23 (83.0 pmol/mg; n = 11) cAMP levels declined again (Fig. 3A). Thus, significantly higher cAMP concentrations were observed at ZT 18 and 20 as compared to ZT 9 (Dunn’s post hoc test, p < 0.05). In contrast to cAMP oscillations, the lowest IP_3_ concentration (Fig. 3B) was detected at ZT 16 (291.5 fmol/mg; n = 16) and the maximum at ZT 18 (565.2 fmol/mg; n = 16). At ZT 18 and ZT 20 (408.3 fmol/mg, n = 33) IP_3_ concentrations were highly significant increased as compared to ZT 9 (333.9 fmol/mg, n = 32, Dunn’s post hoc test, p < 0.01). Furthermore, IP_3_ baseline levels at ZT 18 were significantly higher as compared to ZT 16 (Dunn’s post hoc test, p < 0.05). In contrast, concentrations in cGMP did not differ significantly during the course of the day (Fig. 3C).

### OA elevated cAMP- and IP3 concentrations specifically, dose-dependently, and ZT-dependently

**Fig 4:**
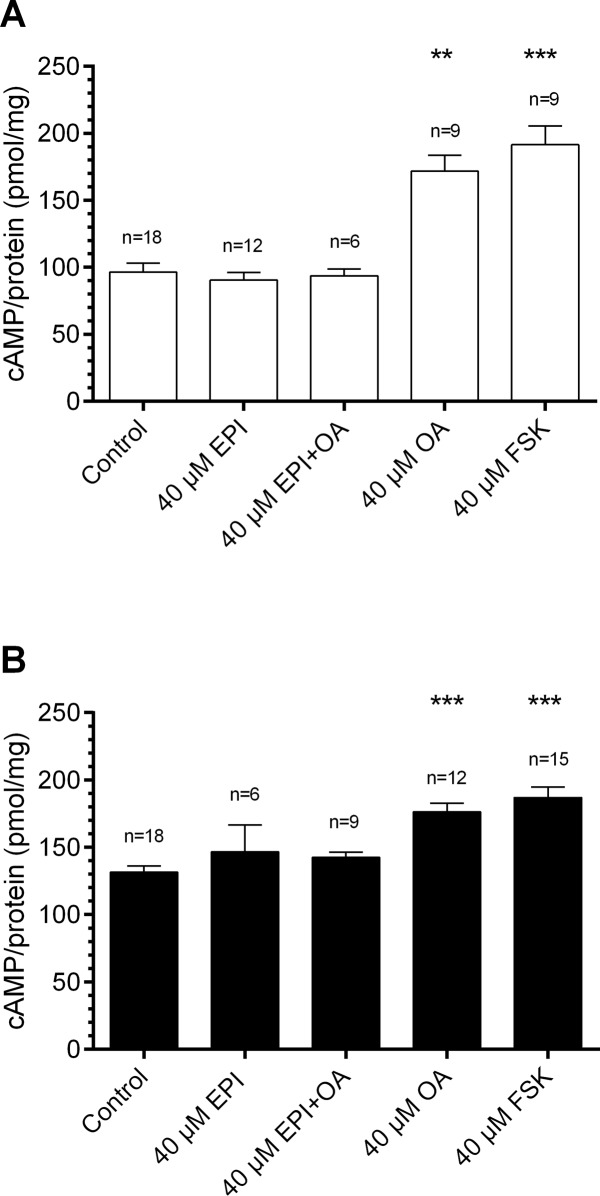
Octopamine (OA) increased cAMP levels in ELISAs of *M*. *sexta* antennae significantly at Zeitgebertime (ZT) 9 (A) and ZT 20 (B). Antennal lysates were stimulated with no drug, 40 μM epinastine (EPI, OA antagonist), 40 μM EPI and OA, 40 μM OA or 40 μM forskolin (FSK, adenylyl cyclase activator), respectively (n = 1 contains 6 antennae). At both ZTs tested 40 μM OA and 40 μM FSK increased cAMP levels highly significant compared to controls (ZT 9: OA, 169.4 pmol/mg, n = 9; FSK, 191.7 pmol/mg, n = 9; ZT 20: OA, 179.8 pmol/mg, n = 12; FSK, 186.9 pmol/mg, n = 15; Dunn’s post hoc test, ** p < 0.01). Co-application of EPI prevented OA dependent cAMP increases. Baseline cAMP levels were higher at ZT20 (A, 131.6 pmol/mg, n = 18) compared to ZT9 (B, 96.3 pmol/mg, n = 18) and could not be reduced via EPI (ZT 20: 146.6 pmol/mg, n = 6; ZT 9: 90.5 pmol/mg, n = 12).

**Fig 5:**
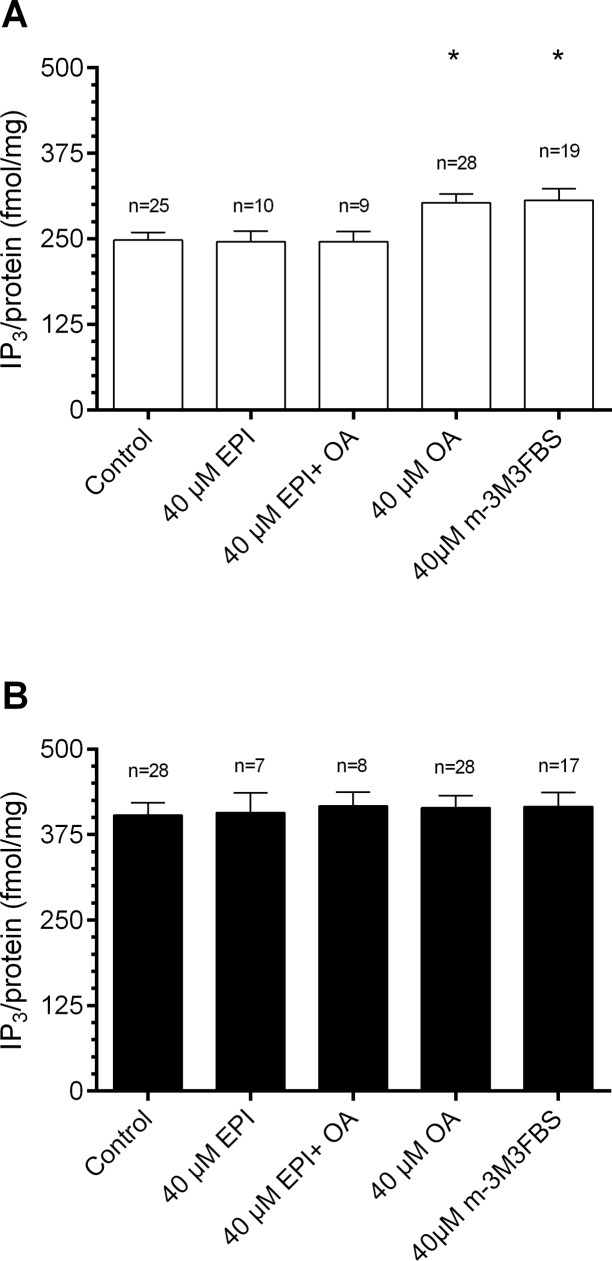
Octopamine (OA) elevated IP_3_ levels in ELISAs at Zeitgebertime (ZT) 9 (A) but not at ZT 20 (B). Antennal IP3 concentrations were quantified with ELISA after incubation with or without (control) 40 μM epinastine (EPI, OA antagonist), 40 μM EPI and OA, 40 μM OA, 40 μM m-3M3FBS (phospholipase C [PLC] agonist), respectively (n = 1 contains 6 antennae). Activation of PLC with OA or its activator both increased IP3 concentrations moderately to the same level (control, 248.2 fmol/mg, n = 25; OA, 302.7 fmol/mg, n = 28; m-3M3FBS, 306.0 fmol/mg, n = 19) at ZT 9 (Dunn’s post hoc test, p < 0.05) but not at ZT 20 (control, 403.7 fmol/mg, n = 28; OA, 414.2 fmol/mg, n = 28; m-3M3FBS, 416.0 fmol/mg, n = 17), where the same maximum level was already obtained in the control.

Additionally, at the moths’ resting (ZT 9)- and activity phase (ZT 20) it was tested whether OA receptors signal via cAMP and/or IP_3_ in the hawkmoth antenna (Figs. 4, 5, Tables 2, 3). First, it was examined whether OA can elevate cAMP levels and whether the OA receptor antagonist EPI is able to prevent OA-dependent second messenger rises. Baseline control cAMP levels were lower at ZT 9 as compared to ZT20, as shown previously (Fig. 3A). Addition of 40 μM OA in the incubation highly significant increased cAMP concentrations as compared to controls at both ZTs tested (ZT 9: 169.4 pmol/mg, n = 9; ZT 20: 179.8 pmol/mg, n = 12; Dunn’s post hoc test, p < 0.01; Fig. 4A,B), to about the same maximal values. Co-application of 40 μM EPI prevented OA dependent cAMP rises (ZT 9: 90.5 pmol/mg, n = 12; ZT 20: 142.7 pmol/mg, n = 9; Fig. 4A,B). Then, it was examined whether adenylyl cyclase activity varies ZT-dependently. Application of the adenylyl cyclase activator FSK (40 μM) at both ZTs highly significant increased cAMP concentrations to 191.7 pmol/mg at ZT 9 and to 186.9 pmol/mg at ZT 20 (Dunn’s post hoc test, p < 0.001; Fig. 4A,B). Additionally to cAMP OA also elevated IP_3_ concentrations in hawkmoth antennae. Both OA and the phospholipase C activator m-3M3FBS elevated IP_3_ levels significantly at ZT 9 (control, 248.2 fmol/mg, n = 25; OA, 302.7 fmol/mg, n = 28; m-3M3FBS, 306.0 fmol/mg, n = 19; Dunn’s post hoc test, p < 0.05), but not at ZT 20 (control, 403.7 fmol/mg, n = 28; OA, 414.2 fmol/mg, n = 28; m-3M3FBS, 416.0 fmol/mg, n = 17; Fig. 5A,B, Table 3). The maximal IP_3_ values in the presence of m-3M3FBS were higher at ZT 20 as compared to ZT 9. The OA receptor antagonist EPI prevented OA-dependent IP_3_ rises at ZT 9 (Fig. 5A) but did not decrease IP_3_ baseline concentrations at ZT 20 (Fig. 5B), which were elevated as compared to ZT 9. Thus, there is at least one other signal with stronger impact compared to OA which elevates IP_3_ levels daytime-dependently and to maximal values at the hawkmoths'activity phase.

**Table 2:** Effects of octopamine (OA), epinastine (EPI) and forskolin (FSK) on cAMP synthesis.

	cAMP ZT 9 (pmol/mg)		cAMP ZT 20 (pmol/mg)	
Concentration [mol/l]	mean±SE	n	mean±SE	n
4 x 10^–10^ OA	96.2±8.0	6	127.3±6.6	4
4 x 10^–9^ OA	114.6±12.8	6	148.1±13.7	13
4 x 10^–8^ OA	128.9±10.7	6	151.6±20.6	6
4 x 10^–7^ OA	130.0±10.1	5	160.1±16.9	5
4 x 10^–6^ OA	145.4±10.8	12	171.3±15.1	17
4 x 10^–5^ OA	169.4±12.4	9	179.8±5.4	12
4 x 10^–4^ OA	172.6±22.6	6	186.6±12.6	6
4 x 10^–5^ EPI	90.5±5.6	12	146.6±20.3	6
4 x 10^–5^ EPI+OA	93.6±5.2	6	142.7±3.8	9
4 x 10^–5^ FSK	191.7±14.0	9	186.9±7.9	15

**Table 3:** Effects of octopamine (OA), epinastine (EPI) and m-3M3FBS on antennal IP_3_ concentrations.

	IP_3_ ZT 9		IP_3_ ZT 20	
	(fmol/mg)		(fmol/mg)	
Concentration	mean±SE	n	mean±SE	n
[mol/l]				
4 x 10^–5^ OA	302.7±12.9	28	414.2±18.2	28
4 x 10^–5^ EPI	245.8±15.4	10	407.4±28.7	7
4 x 10^–5^ EPI+OA	245.7±14.9	9	416.7±20.4	8
4 x 10^–5^ m-3M3FBS	306.0±17.4	19	416.0±20.5	17

### Sensitivity to OA is maximal during the hawkmoth's activity phase

**Fig 6:**
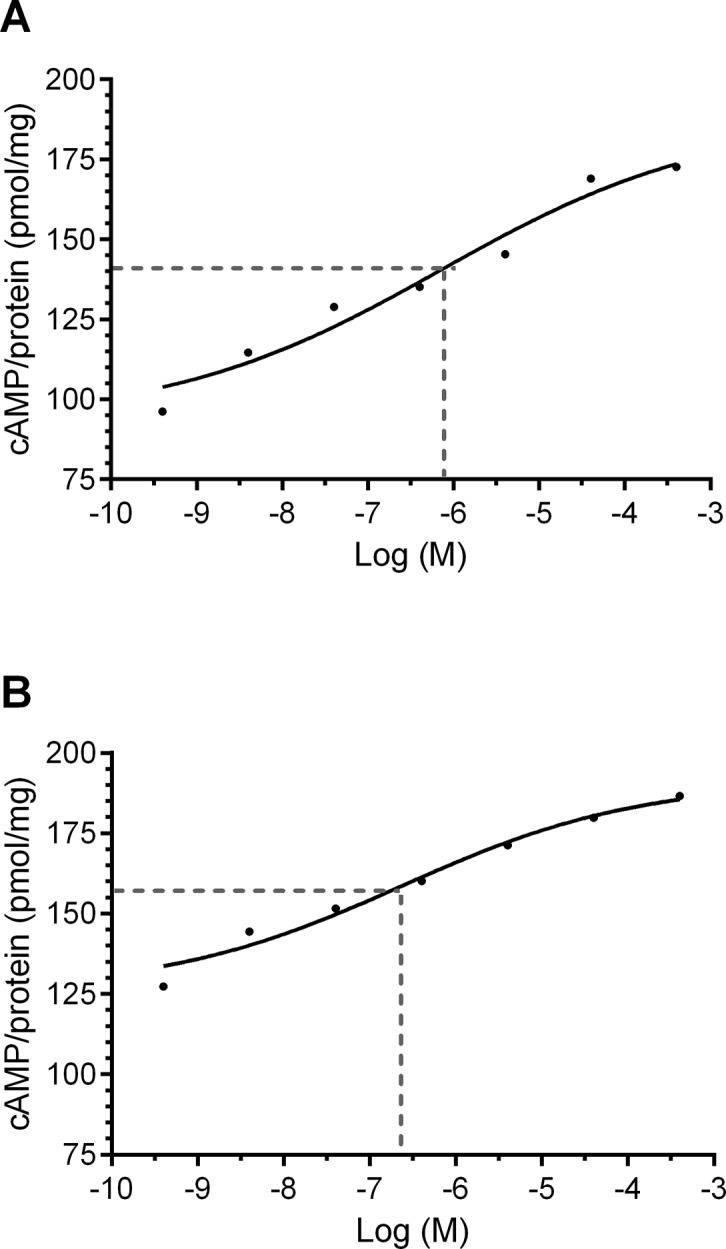
The OA-receptor displays higher sensitivity (= lower threshold) at the activity phase of the hawkmoth. EC_50_ values were lower at Zeitgebertime (ZT) 20 (B) as compared to ZT 9 (A). The log EC_50_ was-6.15 ± 0.85 M at ZT 9 and-6.63 ± 0.48 M at ZT 20 (n = 50 at ZT 9; n = 63 at ZT 20), while calculated peak values were 187.8 pmol/mg cAMP at ZT 9 and 191.2 pmol/mg cAMP at ZT 20. The accuracy of fit was above 0.95.

Next, EC_50_ values of the OA receptor were calculated at both ZTs (Fig. 6A,B). Accordingly, the sensitivity to OA was higher at ZT 20 (EC_50_ = 234.5 nM) as compared to ZT 9 (EC_50_ = 703.5 nM; Fig. 6). In accordance with FSK induced maximal cAMP levels (Table 2), at ZT 9 the calculated maximal value of the EC_50_ equation was 187.8 pmol/mg and at ZT 20 191.2 pmol/mg cAMP,.

### OA elevated antennal cAMP levels within 50 milliseconds

**Fig 7:**
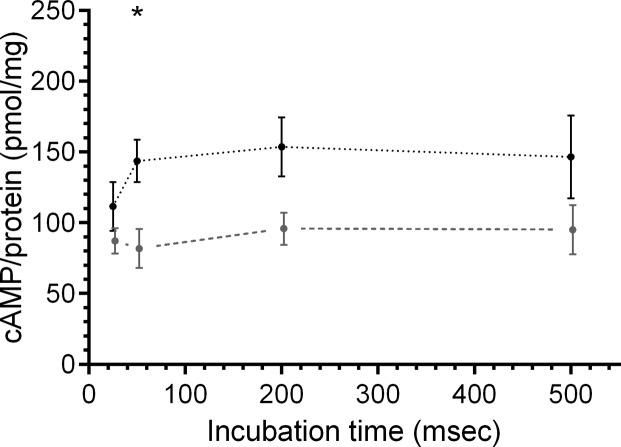
Octopamine (OA) elevated cAMP levels within 50 ms at the animals resting phase. Antennal homogenates were incubated with either control buffer (long stipples, lower line) or with 40 μM OA buffer (short stipples, upper line) for 25, 50, 200 or 500 ms at ZT9. OA significantly increased cAMP levels after 50 ms (Control: 81.8 pmol/mg, n = 9; OA: 143.6 pmol/mg; n = 8) for at least 500 ms (Dunn’s post hoc test, p < 0.05; n = 1 contains 6 antennae).

To determine whether OA receptors signal on a time scale of ms fast kinetic assays (Materials and Method) were developed to quantify OA-dependent cAMP rises (Fig. 7, Table 4). After 25 to 500 ms OA-dependent increases in cAMP concentrations were obtained. Significant rises in cAMP occurred within 50 ms after OA stimulation (143.6 pmol/mg; n = 8; Dunn’s post hoc test, p < 0.05), and remained elevated for durations of at least 500 ms.

**Table 4:** Kinetic of octopamine (OA) induced cAMP increases.

	control		40 μM OA	
	(pmol/mg)		(pmol/mg)	
Incubation time	mean±SE	n	mean±SE	n
[msec]				
25	87.2±8.9	9	111.6±17.3	8
50	81.8±13.8	8	143.6±15.0	8
200	95.9±11.4	8	153.7±20.8	8
500	95.1±17.4	8	146.5±29.1	7

### Centrifugal tyramine-immunoreactive (TA-ir) neurons project from the brain to the sensory cell layer of the hawkmoth’s antenna

**Fig 8:**
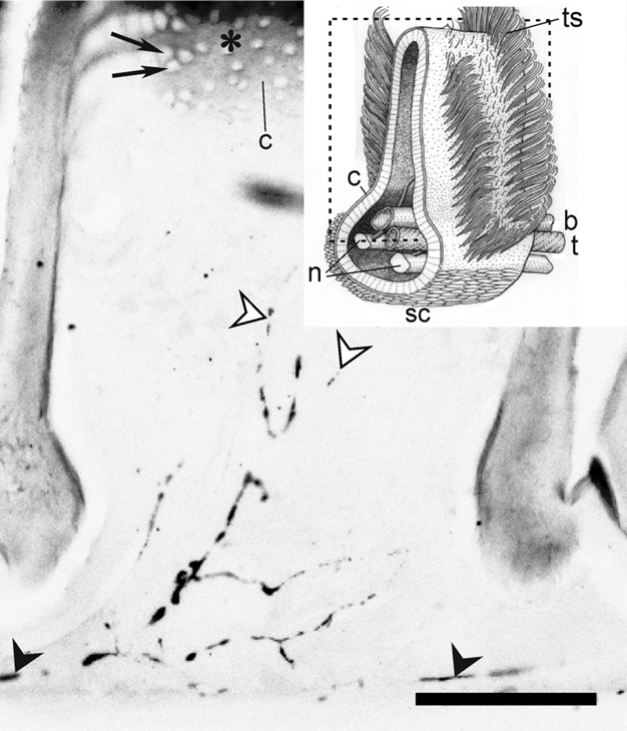
Immunocytochemistry with an antibody against the octopamine precursor tyramine on cryostat-sections of the antennal flagellum of *M*. *sexta* males. Longitudinal cryostat section through an annulus of the antennal flagellum. The insert shows a scheme of a cross-section through the antennal flagellum. Dashed lines indicate the plane of the longitudinal section (modified after [78]. The dark rims of the cryostat sections are the cuticular walls of the annulus. On top the small holes in the cuticle (c, arrows) are cross sections through the cuticular hairs of pheromone-sensitive long trichoid sensilla (ts). The tyramine-immunoreactive fiber (filled arrowheads) projected along the antennal nerve bundle above the trachea at the bottom of the cryostat section. It send a bifurcation up into the sensory epithelium, along the fascicle of sensory nerve axons, cascading a fine meshwork of branches with varicosities (open arrowheads) over the sensory epithelium. The leading edge (asterisk) lies opposite to the scale (sc) side. b blood vessel, c Cuticle, n two nerve bundles per antennal nerve, t trachea,. Scale bar = 100 μm.

Since we were not able to obtain OA-specific staining in the hawkmoth brain or antenna with commercially available OA antisera we employed an antibody against the OA precursor TA to search for biogenic amine-releasing neurons in the antenna (Fig. 8). One TA-ir fiber each was identified in the two fascicles of one antennal nerve. The two fascicles per antennal nerve consist of axons supplying either side of the antenna, innervating mostly olfactory sensilla. Thus, one TA-ir axon which forms a fine web of varicose branches supplies either side of the (in cross-section key-shaped) antennal flagellum. The aminergic axon runs closely associated with the main trachea along the axon bundles of the sensory receptor neurons of the flagellar antennal nerve projecting up and down into the sensory cell layers of sensilla and scale side (Fig. 8). In the sensory cell layer fine varicose arborizations appeared to contact all types of sensilla such as the long pheromone-sensitive trichoid sensilla. However, it could not be determined whether sensory neurons and/or non-neuronal supporting cells of the sensory sensilla were directly contacted by the TA-ir fibers.

## Discussion

With ELISAs it was examined whether daytime-dependent changes in OA and second messenger concentrations in the antenna of the male hawkmoth *M*. *sexta* could be responsible for daytime-dependent changes in pheromone-responsiveness. Indeed, antennal OA concentrations changed during the course of the day with highest concentrations correlating with the moth's activity phase and lowest concentrations correlating with rest. In synchrony with OA concentrations, ZT-dependent changes in the concentrations of cAMP occurred, indicating that OA signals via adenylyl cyclase activation. In addition, ZT-dependent changes in the sensitivity of OA receptors appeared to control ZT-dependent changes in cAMP levels. In contrast, levels of cGMP remained constant throughout the day under conditions of isolation from female pheromone. Since concentration changes of IP_3_ only partly correlated with OA concentration changes next to OA-dependent stimulation of phospholipase C (PLC) also another stimulus is present. Interestingly, IP_3_ concentrations were minimal during maximal feeding at dusk and maximal during the peak of flight activity. Thus, it was suggested that possibly odors present in the environment of moths such as the artificial flowers we offered for feeding might adapt PLC and suppress IP_3_ levels. Furthermore, sensory feedback during active flight might affect PLC activity. In conclusion, we hypothesize that circadian changes of OA in the hemolymph synchronize circadian changes of sensory thresholds in ORNs, which appear to be peripheral circadian pacemakers. In addition, also stimulus-dependent fast OA signaling occurs at the periphery via centrifugal biogenic amine-containing neurons. Possibly, they respond to acute stress signals or they mediate reward signals during olfactory learning which might occur already at the periphery.

### Rhythmic behavior of hawkmoths’ is endogenously generated and synchronized via odors

Similar to the crepuscular hawkmoths in the wild also in isolation *M*. *sexta* males expressed maxima in feeding activity at dusk, while increased flight activity was observed during the night. Even in the absence of females, maximal flight activity occurred in captivity at the late night when males usually perform mating flights in the wild [33]. Accordingly, OA and cAMP levels were significantly elevated during this time, both increasing sensitivity and temporal resolution of pheromone detection [24,34]. Thus, behavioral, electrophysiological, and biochemical data correlated accordingly, hinting a major role for OA in the ZT-dependent control of odor-dependent behavior. However, in the wild, hawkmoth feeding rhythms are phase-shifted for a few hours into the night apparently due to olfactory and visual cues which synchronize moth behavior with nectar production of their food plants [31,35–37]. The preferred plant for hawkmoth feeding is the Solanacea *Datura wrightii* which opens trumpet-shaped flowers at dusk, producing most nectar about 1–2 hours thereafter when maximal feeding by its moth pollinators occurred [38,39]. After feeding, in the second half of the night male moths start searching for females [33]. Since pheromone-sensitivity of olfactory receptor neurons (ORNs) is maximal during the late night, male moths behavioral rhythms are synchronized with rhythms of antennal pheromone-sensitivity. In addition, males are synchronized with their species-specific females which express rhythmic pheromone production [33,34,40]. However, it is still not resolved which circadian clocks and which coupling signals control the rest-activity rhythms of the crepuscular hawkmoths [33,41] (Fig. 2). Furthermore, it remains to be studied which circadian pacemakers and coupling factors synchronize both sexes and respective pollinator-plant interactions.

In the fruit fly *Drosophila melanogaster* circadian rhythms in clock gene expression identified central circadian pacemakers in the midbrain and also peripheral circadian pacemakers such as ORNs in the antenna [42]. Since OA-immunoreactive (OA-ir) neurons were identified in the suboesophageal ganglion with projections into the brain [43,44] we assume that two of these project up into the antenna of the hawkmoth. However, while biogenic amine-ir neurons terminate at the antennal heart in cockroaches [45] in the hawkmoth they terminate in the sensory cell layer of the antennal flagellum. Other OA-ir neurons were described in the ventral nerve cord, which are known to release OA into the hemolymph in several insect species [46–49]. We hypothesize that these OA-ir neurons also express circadian clock genes and release OA clock-controlled as predominant circadian coupling signal into the hemolymph [2,44,50–52]. The circadian rhythm of OA concentrations in the hemolymph then couples central with peripheral circadian clocks in the antenna in synchrony with the external light dark cycles [2,53,54].

### Daytime-dependent rhythms of olfactory sensitivity are endogenously generated in antennal receptor neurons and are coupled with behavioral rhythms also via central coupling factors

Also in the hawkmoth, ORNs appear to be peripheral circadian pacemakers, since they rhythmically express the circadian clock gene *period* [55]. Very likely, this circadian clock work of the ORNs controls the ORNs olfactory sensitivity which expressed daytime-dependent rhythms with maxima during the activity phase of the hawkmoths [33,34], comparably to fruit flies. In *Drosophila* antennae circadian rhythms of clock gene expression were required for the expression of circadian rhythms in olfactory sensitivity [56–58]. Also, in the Madeira cockroach circadian rhythms were observed in pheromone-sensitivity in the antenna [7,59]. Accordingly, the Madeira cockroach *Rhyparobia* maderae expresses synchronized mating activity of both males and females [6]. However, unexpectedly, mating rhythms were not synchronized with olfactory sensitivity rhythms in the antenna but these were mediated via centrifugal control from the brain [7,59]. Since we detected circadian oscillations in antennal cAMP levels in synchrony with behavioural rhythms most likely underlying synchronized peripheral rhythms were masked by sensory adaptation [29]. Thus, in conclusion, circadian rhythms of olfactory sensitivity are generated endogenously via antennal circadian clocks in the ORNs. The rhythms are phase-controlled and synchronized via centrifugal control of central circadian pacemakers [7,29,60]. One of these central circadian pacemakers appear to be octopaminergic neurons which rhythmically release OA as central coupling factor into the hemolymph.

### The stress hormone OA signals via cAMP and IP_3_

Two general types of G-protein-dependent OA receptors are known [8,26,61]. The α-adrenergic-like OA receptor (OctαR) modulates intracellular Ca^2+^- as well as cAMP levels. In contrast, activation of β-adrenergic-like OA receptors (OctβR) only affects cAMP levels [26]. In addition, also a third type of OA receptor is known which also binds tyramine [26]. So far, we provided evidence for specific OctαR-type receptors in hawkmoth antennae which could be blocked by EPI. Since a previous study only found evidence for one putative OA receptor in hawkmoths it is possible that this is the only OA-receptor present [62]. Its amino acid sequence expressed a high degree of homology to OA receptors from the honey bee *Apis mellifera* and the American cockroach *Periplaneta americana* which are also OctαR types [62–64]. While most of the known OA receptors were expressed in mammalian cell lines and showed an effective dose for OA in the low micromolar range [63–66], only *D*. *melanogaster* DmOA2 showed a higher affinity with an EC_50_ at 3 x 10^–8^ M [61]. Thus, EC_50_ values of *M*. *sexta* OA receptors are consistent with previous studies in other insects. However, since IP_3_ rhythms and cAMP rhythms were not phase-coupled, we either have also OctβR present in hawkmoth antennae, and/or additional signals control IP_3_ levels in the insect antennae, such as odors which stimulate an odor-dependent signal transduction cascade.

### Pheromone transduction is second messenger-dependent and is modulated via the stress hormone OA Zeitgebertime-dependently

Insect odor transduction is still under debate. One hypothesis suggests that olfactory receptors (ORs) together with the conserved ion channel ORCO underlie an ionotropic signal transduction cascade [67]. Alternatively, either a sole metabotropic or a mixed ionotropic and metabotropic odor transduction cascades were suggested [28,68–71]. In the hawkmoth *M*. *sexta* so far, no evidence for an ORCO-based ionotropic signal transduction cascade was found [70]. ORCO was suggested to be a hormone-controlled pacemaker channel controlling spontaneous activity and, thereby, threshold and temporal resolution of pheromone detection [28,71]. In moths, pheromone-receptors appear to couple to phospholipase Cβ, increasing IP_3_ levels resulting in Ca^2+^ channel opening [28,72–74]. So far, pheromone-dependent rises of cAMP were not observed in moth antennae, but adapting pheromone concentrations caused slow, sustained rises in cGMP levels which correlated with processes of odor-dependent adaptation [74–76]. Since in contrast to a former study with cockroaches [29] in the current study the males were isolated from their females, they were not exposed to adapting concentrations of female pheromone. Thus, cGMP levels remained constantly low over the course of the day.The observation of maximal IP_3_ baseline levels before OA maxima, while moths were flying is consistent with an IP_3_-dependent odor transduction cascade in *M*. *sexta*. Co-application of OA during moth pheromone detection appears to increase the sensitivity and temporal resolution of pheromone detection via activation of IP_3_- and pheromone-dependent ion channels [21,22,24,28]. Elevations of cAMP might further boost sensitivity and temporal resolution of the pheromone transduction cascade of the hawkmoth via activation of cAMP-dependent transient Ca^2+^ channels [34,77]. Alternatively or concurrently, OA-dependent second messenger changes might affect opening probability of the pacemaker channel ORCO, since OA-receptor antagonists deleted spontaneous activity of ORNs [24].

**To summarize,** future experiments will challenge our hypothesis that circadian release of OA into the hemolymph is mediated via octopaminergic circadian pacemakers in the ventral nerve cord. These circadian changes of OA in the hemolymph synchronize circadian rhythms of odor detection thresholds, generated endogenously via the ORNs as peripheral circadian clocks in the antenna. In addition, these circadian rhythms in pheromone detection thresholds are synchronized via other coupling signals such as pheromones. The pheromones as interspecific coupling signals guarantee optimized mating behaviour of both sexes since they synchronize the male behavior with the physiological rhythms of female reproduction. In addition, octopaminergic centrifugal neurons which relay acute stress-signals or reward signals might overrule circadian rhythms via fast and phasic release of second messengers which affect odor sensitivity in the context of learning and memory at the periphery.
